# The effect of ad hominem attacks on the evaluation of claims promoted by scientists

**DOI:** 10.1371/journal.pone.0192025

**Published:** 2018-01-30

**Authors:** Ralph M. Barnes, Heather M. Johnston, Noah MacKenzie, Stephanie J. Tobin, Chelsea M. Taglang

**Affiliations:** 1 Psychology Department, Montana State University, Bozeman, Montana, United States of America; 2 Psychology Department, Columbus State Community College, Columbus, Ohio, United States of America; 3 Department of Social Sciences, Clermont College, University of Cincinnati, Batavia, Ohio, United States of America; 4 School of Psychology, Australian Catholic University, Banyo, Queensland, Australia; 5 Psychology Department, Hood College, Frederick, Maryland, United States of America; University of Illinois-Chicago, UNITED STATES

## Abstract

Two experiments were conducted to determine the relative impact of direct and indirect (ad hominem) attacks on science claims. Four hundred and thirty-nine college students (Experiment 1) and 199 adults (Experiment 2) read a series of science claims and indicated their attitudes towards those claims. Each claim was paired with one of the following: A) a direct attack upon the empirical basis of the science claim B) an ad hominem attack on the scientist who made the claim or C) both. Results indicate that ad hominem attacks may have the same degree of impact as attacks on the empirical basis of the science claims, and that allegations of conflict of interest may be just as influential as allegations of outright fraud.

## Introduction

It might appear that scientific findings have been at the center of a number of hot-button issues such as the safety of childhood vaccinations, pharmaceutical compounds and genetically modified crops. However, the credibility of those making the science claims might be at least as important as the science behind the claims. According to Bromme and Goldman [1] the complexity inherent in many science claims means that lay persons may not be able to directly evaluate science claims. Instead, non-scientists may have to resort to second-hand evaluation. They claim that most people may have to first determine *who* to trust before they can determine *what* is true. Consistent with this, Brossard and Nisbet [2] have shown that deference to the source of science claims is an important component in understanding public attitudes regarding agricultural biotechnology. Metzger, Flanagin, and Medders [3] found that people frequently lack either the time or cognitive resources to evaluate science information systematically. For this reason, Metzger et al. found that individuals often rely on heuristics to determine the credibility of those making the science claims.

Source credibility is a positive characteristic that may increase the likelihood that a person will be influenced by a message [4]. Source credibility can include such factors as expertise [5] and bias [6,7]. Trust is similar to source credibility, but also includes an aspect of risk [8,9]. Mayer, Davis, and Schooman [9] clarify the relevance of risk to trust by noting that trust implies that the person doing the trusting has something to lose if the communicator is lying. According to Mayer et al. [9] trust has three components: ability, benevolence, and integrity. Respectively, these components require that the person given trust A) has the aptitude and experience to fulfill the trust, B) has a desire to fulfill the trust, and C) adheres to a set of acceptable moral principles. It is clear that the source credibility and trust are overlapping constructs [9–12]. A key distinction between the two constructs is that source credibility is more narrowly related to the topic of persuasion, whereas trust may related to other aspects of behavior. Generally speaking, an individual that is credible will also be trusted, and vice versa.

Numerous studies have shown that scientific information may not have as much impact on the public’s attitude as trust in scientists and government policy-makers [13–15]. Given the evidence for a link between trust and public opinion, cases of fraud and misconduct, and conflicts of interest may play a powerful role in shaping the public’s trust in scientists and the ability of scientists to influence the public. The popular media sometimes covers stories involving scientific incompetence (e.g. the Fleischmann and Pons affair) and fraud and/or misconduct committed by scientists [16–18]; and there is no shortage of reporting on scientists with conflicts of interest [19–22]. Personal email communications of several climate scientists were leaked to the public in 2009 in an event known as “climategate”. These email communications revealed that scientists sometimes engage in ad hominem attacks on their peers, and knowledge of these emails likely led some to distrust the authors of these emails [23].

There is wide agreement in regards to the importance of trust in the fields of risk analysis and public understanding of science (PUS) [13,15,24–27]. This research shows that, in the absence of trust in government and related institutions, imparting knowledge to the public is insufficient to change public opinion [14,28]. Trust in scientists has been implicated as an important factor in acceptance of genetically modified foods [29], irradiation of foods [24], stem cell research [30,31], nanotechnology [32,33], and the creation of a nuclear waste storage facility [26]. Additionally, there are several studies [34,35] that implicate financial ties as an important component related to acceptance of science claims.

Although we are interested in factors that reduce the public’s confidence in science claims, we are not concerned with the issue of trust *per se*. Rather, our focus is on the specific methods that can be used to attack and undercut science claims and the relative effectiveness of those methods. One method for attacking a science claim is a direct attack on the empirical foundation of the claim. The ad hominem attack is a more indirect method for attacking a science claim. Here we are concerned with three forms of ad hominem attack: allegations of misconduct, attacks directed at motives, and attacks directed at competence. Seen through the lens of the Mayer et al. model [9], misconduct and motive-related attacks are related to benevolence and integrity, while attacks directed at competence are related to ability. Allegations of misconduct are commonly reported in widely read journals such as *Science* [36,37] and these allegations are sometimes reported on by the media [18,38,39]. Ad hominem attacks focused on motivation (often in regards to conflicts of interest) have appeared in peer reviewed research articles [40,41], books written for popular audiences [42–44] and the mass media [19,20]. A third type of ad hominem attack, attack on the competence of researchers, is less common. Articles that undergo peer review rarely include such attacks and examples of such attacks are not commonly found in mainstream mass media. It is, however, possible to find examples of such attacks. In 2003 the *British Medical Journal* published a controversial article on second hand smoke [45] and because of the controversy surrounding the article, the editor of the journal chose to post many of the email responses to the article on-line and some of these responses were published in the print version of the *British Medical Journal* as well [46]. As noted by the associate editor of BMJ, the rapid responses were plagued with ad hominem attacks and some of the adjectives used in these attacks (e.g. comic and crappy) would almost certainly not be found in the peer reviewed literature.

Ad hominem attacks have the potential to be both fallacious and effective. In regards to the first point, ad hominem attacks have been described as fallacies of argumentation when the issue of an opponent’s character is not relevant to the issue being discussed [47–49]. However, accusing a scientist of misconduct, conflicts of interest, or a general lack of competence might be relevant to the claims advanced by that scientist, and therefore a case can be made that ad hominem attacks are not necessarily fallacies [50–53]. Regarding the issue of effectiveness, it should be noted that even in the case that an ad hominem attack is considered fallacious, it still may be effective [35,47,49,54–59]. Macagno [60] suggests a mechanism for the effectiveness of ad hominem attacks: they challenge the authority of a speaker and thereby undercut arguments that were seen as credible due to the expertise of the speaker.

In the current studies we presented participants with science claims that were attributed to particular scientists, followed by information that attacked either A) the empirical data upon which the initial claim was based or B) the researcher who generated the empirical data that supported the claim or C) both the empirical data and the researcher. For our purposes, we used attitude towards science claims to measure the impact of the various kinds of attacks directed against the claims. The basic procedure and attitude-based dependent measure used in the present studies are nearly identical to those used by Barnes, Tobin, Johnston, MacKenzie, and Taglang [61]. We chose the procedure and dependent measure used by Barnes and colleagues because they found that their attitude measures were in agreement with a choice measure. Specifically, participants in the final experiment chose a prescription drug in a manner consistent with the attitude measures used in the first four experiments. We felt that if we used an attitude measure that is known to agree with a choice measure, then we could assume that our results would have practical relevance. Experiments 1 and 2 used within-subjects designs to examine the influence of six different types of attack. The attack in the *empirical* condition was directed exclusively towards the empirical foundation upon which a claim was based. The attacks in *the past misconduct*, *conflict of interest*, *education*, and *sloppy* conditions were examples of ad hominem attacks that had potential to erode trust and source credibility. The past misconduct condition is an example of a claim about researcher misconduct and is related primarily to the integrity and benevolence aspects of trust. The conflict of interest attack is directed at the motives of the researcher and is also related to the integrity and benevolence aspects of trust. The education and competence attacks are directed at the researcher’s competence and are related to the ability aspect of trust. In the final condition, *relevant misconduct*, the attack was directed both against the data upon which a claim was based and against the scientists making the claim. For this reason, the relevant misconduct attack has both an ad hominem aspect (related to the benevolence and integrity aspects of trust) and an aspect that directly calls into question the data. In the empirical condition the data, but not the researcher, was a target of attack, whereas in the relevant misconduct condition both the data and researcher were attacked.

We predicted that the greatest degree of attitude change would occur in the relevant misconduct condition because, in this condition, both the researcher and the data are explicitly criticized. We predicted that the second greatest degree of attitude shift would be associated with the empirical condition because the data upon which the claim was based was described as flawed and, unlike ad hominem attacks, attacks on the empirical foundation of a claim are always and obviously relevant. The other four conditions (past misconduct, conflict of interest, education, sloppy) included only ad hominem attacks, therefore we predicted that each of these conditions would have a negative impact on attitude, but that the degree of attitude change associated with these conditions would not be as great as that associated with the other two conditions.

## Experiment 1

### Method

#### Ethics statement

Ethics approval for both studies was obtained from all institutions at which data was collected. For Study 1, data was collected and IRB approval was granted from Columbus State Community College, Lafayette College, Salem State University, University of Cincinnati-Clermont College, and the University of Houston. For Study 2, IRB approval was granted by Lafayette College.

In all cases a written information sheet or a computer screen containing the information was provided to participants, but the committees waived the need for written informed consent from the participants as the research was low risk. Some minor deception was necessary to test our hypotheses, participants' rights and welfare were not adversely affected, and a full debriefing was provided at the end of the study.

#### Participants

The participants comprised 480 undergraduate student volunteers from two community colleges, a private research university, a private liberal arts college, and a state college. The data from twenty participants who failed to finish the questionnaire and from 10 participants who skipped one or more items in the questionnaire were discarded. Data from an additional 11 participants were discarded for failure to follow instructions. The average age of the remaining 439 participants was 24.1 and the sample included 312 women. All participants were enrolled in psychology courses and were given extra-credit for their participation.

#### Stimuli

The initial section of each of eight questionnaire variants contained a series of 24 science claims and the final section included several demographic questions. Twelve of the science claims were distractor items whose function was to keep participants from detecting the purpose of the study. These distractor items were similar to the critical items in that half the distractor items were science claims in isolation and half the distractor items were science claims paired with additional information. The additional information included in half of the distractor items did not include any challenges of the credibility of the source of the claims nor criticism of the research upon which claims were based. The remaining 12 items all contained a science claim (e.g. a falsifiable claim about the natural world) with nine claims related to the topics of bio/medicine and three claims related to energy/climatology (see S1 Table). In order to guarantee that subjects would not have firmly held ideas about these topics and hypotheses, the phenomena mentioned in the questions were either fictions generated by the authors or references to phenomena that would be unfamiliar to participants. In this respect, the stimuli were intended to function like the “blank predicates” commonly used by psychologists interested in deductive reasoning.

Each of the science claims was attributed to a specific researcher, and in each case the researcher’s name was preceded by the descriptor “Dr.” Of the twelve critical items in each questionnaire, six contained a science claim presented in isolation. For the remaining six items, the science claim was followed by an additional sentence that contained additional information that attacked the researcher and/or science. The additional information either pointed out a flaw in the initial research (*empirical condition*) or contained an ad hominem attack on the researcher who made the claim (past misconduct, conflict of interest, education, sloppy) or both (relevant misconduct). Table 1 presents an example of all six types of additional information for one of the topics (see S1 File for the additional information for all 12 claims). In the empirical condition, participants were informed that the research was flawed by a confound, or contained some threat to the internal or external validity. In the relevant misconduct condition, participants were informed that the scientist had fabricated data for the study on which the claim was based. In the past misconduct condition participants were informed that the scientist had fabricated data from an earlier study that s/he had conducted. In the conflict of interest condition participants were informed that the scientist’s findings were favorable towards a source of financial conflict of interest. In the education condition participants were informed that the scientist earned her/his doctoral degree from an institution known to have low standards. In the sloppy condition participants were informed that the scientist was considered by her/his peers to be a sloppy researcher.

**Table 1:** Example of science claim #7 along with all six types of additional information.

Description		Wording used in questionnaire
Initial Claim # 7		According to Dr. Martinez at the University of Oklahoma, dibutylphthalate, a chemical used in Gold Bond foot powder, decreases the risk of some kinds of cancer.
Type of Additional Information	Empirical	Dr. Martinez’s research on Gold Bond foot powder failed to employ a control group with which to compare the cancer rates of his foot powder using group.
	Relevant misconduct	Recently a team of investigators from the National Science Foundation’s ethics committee found that Dr. Martinez fabricated some of the data in his published research on Gold Bond foot powder.
	Past misconduct	Recently a team of investigators from the National Science Foundation’s ethics committee found that Dr. Martinez fabricated some of the data in one of his earlier papers.
	Conflict of interest	Dr. Martinez has been a paid consultant for Chattam, Inc. (the company that makes Gold Bond foot powder) for over 8 years.
	Education	Dr. Martinez received his advanced degree from a university with a reputation for having very low standards.
	Sloppy	Many of the researchers in Dr. Martinez’s field feel that he is a sloppy researcher.

Eight questionnaire variants were created and each contained the same 12 science claims in the same order (see S2 Table for the sequence of items in all 8 questionnaire variants and see S2 File for a sample questionnaire). In all questionnaire variants, 6 of the claims were presented in isolation and each variant contained 1 claim from each of the 6 information conditions (empirical, relevant misconduct, past misconduct, conflict of interest, education, and sloppy). No two questionnaire variants contained the same pairing of science claim with level of the independent variable. Ninety-seven participants completed a paper-and-pencil version of the questionnaire, the remaining participants completed internet-based questionnaires created with Opinio software. The internet-based questionnaires were nearly identical to the paper-and-pencil versions.

After each item (isolated science claim or science claim paired with additional information), respondents indicated their attitude towards the claim using a 6 point scale that was anchored with strongly favor (1) and strongly oppose (6). The instructions and an example provided to participants clearly indicated that responses should reflect attitude towards the truth of the claim itself rather than attitude towards the researcher or the manner in which the research had been carried out. It seemed likely that participants would not find each of the 12 initial science claims equally compelling. Therefore, the dependent measures of interest on trials in which an initial claim was followed by additional information are difference scores rather than the raw attitude scores. In order to calculate difference scores, mean attitude scores for each of the 12 topics were first calculated. For each paired information trial (one containing both the initial claim and additional information), the attitude score was subtracted from the mean score of the appropriate initial claim in isolation (baseline attitude). Negative difference scores indicate that participants found the initial science claims to be less convincing when they were followed by additional information. For instance, across all participants presented with the baseline version of claim number 2, the average attitude was 3.03. If a participant responded with a “4” on the 1–6 scale for claim 2 on a trial in which the claim 2 was followed by additional information that the scientist who made the claim has a reputation for being a sloppy researcher, then that participant’s difference score would be -0.97, indicating a shift away from the average baseline attitude of nearly one integer on the 6 point scale.

### Results

**Fig 1:**
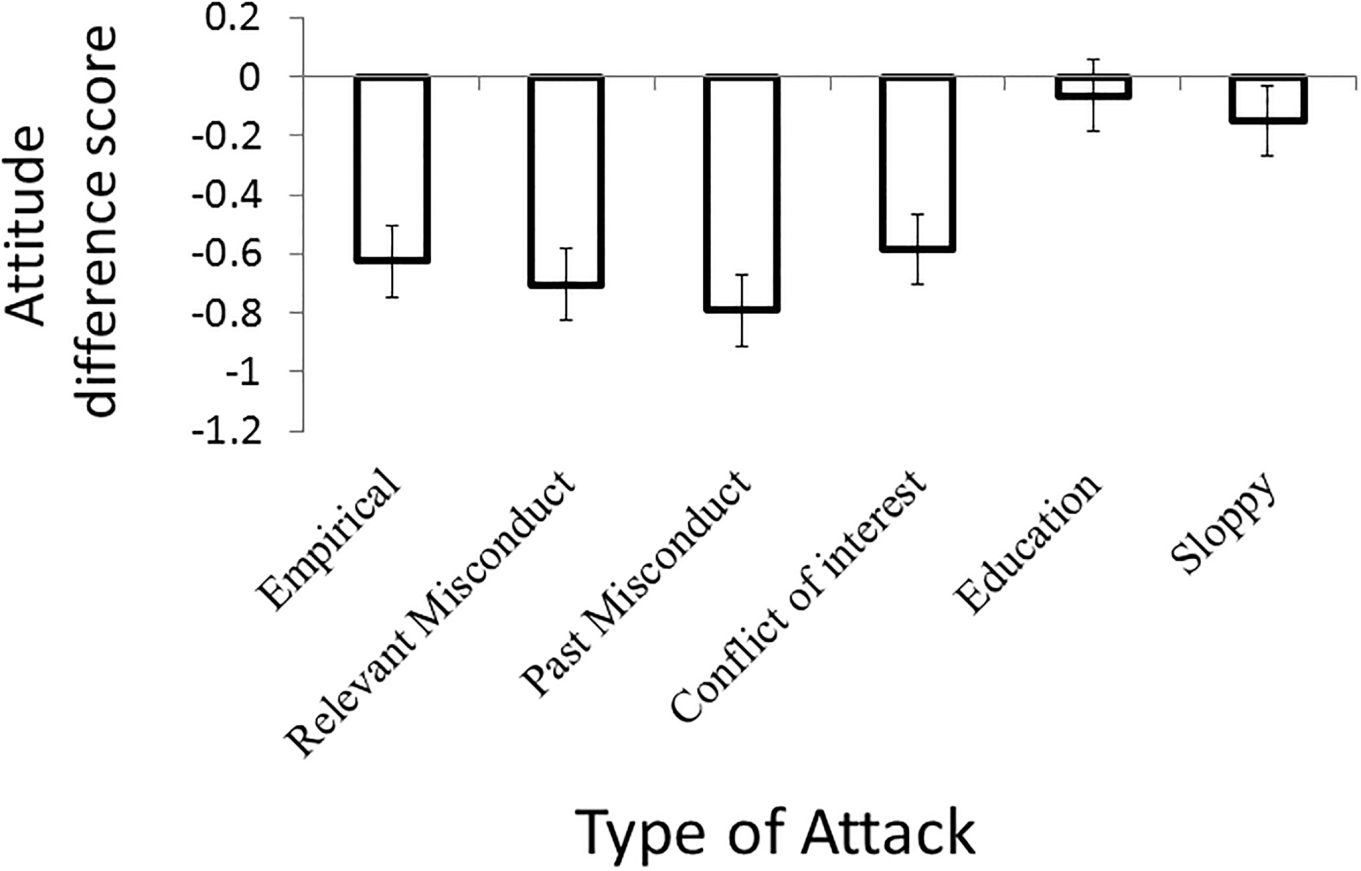
Average attitude difference scores for Experiment 1 as a function of type of additional information (empirical, education, sloppy, relevant misconduct, past misconduct, conflict of interest) collapsed over all 12 science topics. Error bars indicate 95% confidence intervals.

The raw data for Experiment 1 can be found in S3 File. A preliminary analysis of the attitude difference scores considering both gender and age failed to find any significant effects involving either subject variable (*p* > 0.05 in both cases). For this reason, and because there were no theoretical reasons to expect that responses would be influenced by these subject variables, all reported analyses involve data in aggregate form. A within-subjects analysis of variance (ANOVA) of the difference scores for the six conditions revealed a significant effect of additional information, *F*(5,2190) = 25.4, *p* < .001, η^2^ = .06 (see Fig 1). Post-hoc paired samples *t* tests revealed that the difference scores for the education and sloppy conditions were not significantly different from each other (*p* = .3), however, these two conditions were significantly different from the other four conditions (*p* < .001 in all cases). Post-hoc paired samples *t* tests also revealed that the empirical, relevant misconduct, past misconduct, and conflict of interest conditions did not differ from one-another (*p* > .02 in all cases). For all post-hoc tests, the pair-wise alpha level was adjusted in order to maintain a family-wise error rate of .05.

In order to determine if the attitude for each condition was negatively impacted by the additional information, we conducted six single-sample *t* tests comparing the mean difference score of each condition to zero (no effect). These analyses indicated that only the education and sloppy conditions were not significantly different from zero (Table 2). The other four conditions did differ from zero, indicating that the additional information had a negative impact on attitude towards the science claims. As measured by Cohen’s *d*, the empirical, relevant misconduct, past misconduct, and conflict of interest conditions were all characterized by a moderate effect size. Finally, we computed the post-hoc statistical power for these comparisons to be 1, far in excess of the power of .8 frequently recommended for social science research.

**Table 2:** Mean difference scores in Experiment 1 for each condition and results of single-sample *t* tests comparing means to zero.

Type of additional information	*M(SD)*	Single-sample *t* test results
Empirical	-.63(1.43)	*t*(438) = 9.16, *p* < .001, *d* = .44*
Relevant misconduct	-.70(1.39)	*t*(438) = 10.66, *p* < .001, *d* = .51*
Past misconduct	-.79(1.36)	*t*(438) = 12.25, *p* < .001, *d* = .58*
Conflict of Interest	-.59(1.41)	*t*(438) = 8.67, *p* < .001, *d* = .41*
Education	-.07(1.31)	*t*(438) = 1.04, *p* = .3, *d* = .05
Sloppy	-.15(1.35)	*t*(438) = 2.3, *p* = .022, *d* = .11

*significant using Bonferroni family-wise error adjustment which set per-comparison alpha to .008.

## Experiment 2

We had predicted that an attack on the data upon which a claim was based coupled with an ad hominem attack (relevant misconduct condition) would more effectively impact attitude than only an attack on the data (empirical condition). We also predicted that all four strictly ad hominem attack conditions would be associated with less attitude change than either of the two conditions in which the data was explicitly attacked. In contrast to our first prediction, the results of Experiment 1 indicated that the attitude change associated with the empirical condition was no different than that associated with the relevant misconduct condition. Additionally, the same degree of attitude change associated with the empirical and relevant misconduct conditions was also found in two of the strictly ad hominem conditions (past misconduct, conflict of interest). The two ad hominem attacks that addressed the competence of the researcher (sloppy and education) had no effect on attitude. These results are useful in that they reveal the relative effectiveness of various methods of attacking research. However, the sample (college students enrolled in psychology courses) was rather homogenous and not representative of the general population. We can place more confidence in the findings of Experiment 1 if the findings are replicated by another study. To this end, Experiment 2 is an attempted replication of Experiment 1 using a more representative sample of adults. Given the results of Experiment 1, we discard our initial predictions. Our prediction for Experiment 2 is simply that it will replicate the findings of Experiment 1.

### Method

#### Participants

Two-hundred and twenty-four participants were recruited through an opt-in internet panel managed by a survey research firm (Marketing Systems Group). Data from 21 participants were excluded because the participants completed the survey in less than 4 minutes. Data from 3 respondents who chose not to respond to 1 or more items was discarded, and data from 1 respondent was excluded for failure to follow instructions. The remaining participants comprised 199 non-institutionalized adults living in the U.S. The age of respondents ranged from 23 to 83 with a mean of 48.5 and median of 47. Thirty-nine states were represented in the sample and 47.4% of the respondents were female. Nearly 77% of the respondents identified themselves as non-Hispanic white, while 13.8% and 9.2% identified themselves as black and Hispanic, respectively. Additionally, 40.4% of respondents had earned at least one college degree, and 46.2% of the respondents were from households with an annual income below $50k per year. All participants were paid $2.75 for their participation.

#### Stimuli and procedure

The stimuli and procedure of Experiment 2 were identical to that of Experiment 1 with two exceptions. All participants in Experiment 2 responded to an on-line version of the survey (the same Opinio user interface was employed). In addition to age and gender, participants in Experiment 2 were asked to indicate their annual household income, highest level of education attained, and knowledge in regards to science (not very, somewhat, moderately, very knowledgeable).

### Results

The raw data for Experiment 2 can be found in S4 File. A preliminary analysis of the attitude difference scores considering gender, age, knowledge, income, and education failed to find any significant effects involving any of the subject variables (*p* > 0.07 in all cases). Because we found no significant effects of any of our subject variables, and because there were no theoretical reasons to expect that responses would be influenced by these subject variables, all additional analyses were conducted on the data in aggregate. We wanted to conduct an *a priori* power analysis to determine the number of participants we would need to determine if any of the six conditioned differed from zero. To that end, we conducted a power analysis of a two-tailed, single-sample t-test using a Bonferroni corrected alpha level of .0083, and a power of .95. We considered a medium effect size (*d* = .5) to be the smallest meaningful effect. This *a priori* power analysis revealed that we would need 77 participants in Experiment 2. Consistent with the *a priori* power analysis, a post-hoc power analysis revealed that Experiment 2 had an effective power of 1.

**Fig 2:**
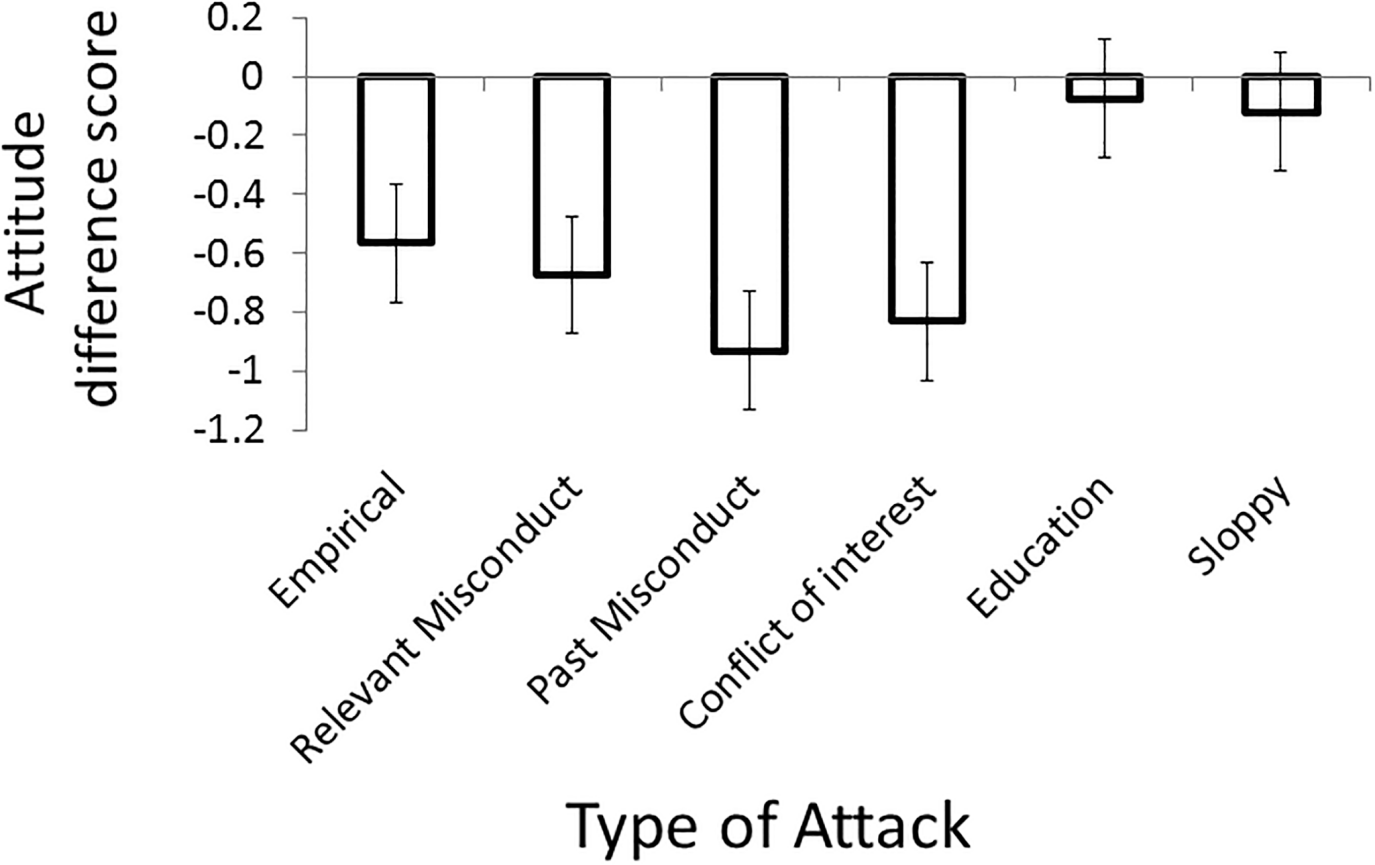
Average attitude difference scores for Experiment 2 as a function of type of additional information (empirical, education, sloppy, relevant misconduct, past misconduct, conflict of interest) collapsed over all 12 science topics. Error bars indicate 95% confidence intervals.

A within-subjects ANOVA of the difference scores for the six conditions revealed a significant effect of additional information, *F*(5, 990) = 14.62, *p* < .001, η^2^ = .07 (see Fig 2). As in Experiment 1, post-hoc paired-samples *t* tests revealed that the difference scores for the education and sloppy conditions were not significantly different from each other (*p* = .74), however, these two conditions were significantly different from the other four conditions (*p* < .003 in all cases). Post-hoc paired-samples *t* tests also revealed that the empirical, relevant misconduct, past misconduct, and conflict of interest conditions did not differ from one-another (*p* > .03 in all cases).

In order to determine if the attitude for each condition was negatively impacted by the additional information, we conducted six single-sample *t* tests comparing the mean difference score of each condition to zero (no effect). As in Experiment 1, these analyses indicated that only the education and sloppy conditions were not significantly different from zero (see Table 3). The other four conditions did differ from zero, indicating that the additional information had a negative impact on attitude towards the science claims. As in Experiment 1, the empirical, relevant misconduct, past misconduct, and conflict of interest conditions were all characterized by a moderate effect size, and all *t* tests were characterized by a statistical power of 1.

**Table 3:** Mean difference scores in Experiment 2 for each condition and results of single-sample *t* tests comparing means to zero.

Type of additional information	*M(SD)*	Single-sample *t* test results
Empirical	-.57(1.46)	*t*(198) = 5.48, *p* < .001, *d* = .39*
Relevant misconduct	-.67(1.34)	*t*(198) = 7.11, *p* < .001, *d* = .5*
Past misconduct	-.93(1.43)	*t*(198) = 9.17, *p* < .001, *d* = .65*
Conflict of Interest	-.83(1.44)	*t*(198) = 8.15, *p* < .001, *d* = .58*
Education	-.07(1.30)	*t*(198) = .8, *p* = .43, *d* = .06
Sloppy	-.12(1.44)	*t*(198) = 1.18, *p* = .24, *d* = .08

*significant using Bonferroni family-wise error adjustment which set per-comparison alpha to .008.

## Discussion

Neither of our main predictions for Experiment 1 were supported by the data. For instance, we found that combining ad hominem attacks with direct attacks on the empirical foundation of the claim was no more effective than an empirical attack in isolation. In contrast to our second prediction, Experiment 1 revealed that some strictly ad hominem attacks (specifically the conflict of interest and past misconduct attacks) are just as effective as attacks on the empirical foundation of a claim. Our only prediction for Experiment 2 was that the result of Experiment 2 would replicate those of Experiment 1, and that prediction was confirmed. The similarity between the results of Experiments 1 and 2 increased our confidence in the pattern of results we found in Experiment 1. The results of Experiment 2 were based on a sample that, relative to Experiment 1, was much more representative of the US population. This indicates that our findings are not specific to a college student population.

As expected, information that a study was critically flawed was associated with negative attitude change towards a claim based on that study. What was not expected was that an ad hominem attack (in the form of an accusation of misconduct) coupled with an explicit attack on the research itself was no more influential than an attack on the research alone. However, at the time we generated our predictions we were not familiar with a study by Luo, Lou, Schatzberg and Sia [62], that found that the effect of message persuasiveness and source credibility were substitutive rather than additive. So while we found the result to be unexpected, the result is consistent with, and a replication of, previous research.

Another failed initial prediction was that strictly ad hominem attacks would not lead to as much attitude change as attacks on the empirical foundation of the science claim. Information about misconduct had an equivalent influence, regardless of whether the misconduct was in regards to the claim in question or was in regards to unrelated research that had been conducted by the scientist in the past. Ad hominem attacks that only mention conflicts of interest had just as great an impact on attitude as claims of misconduct or direct attacks on the research itself. Finally, the two types of additional information that are least likely to appear in peer reviewed or popular sources (i.e. the education and sloppy conditions) did not have a negative impact on attitude.

Mayer et al. [9] claimed that the three components of trust are ability, benevolence, and integrity, and their framework may explain our failed predictions for Experiment 1. In the context of their framework, allegations of misconduct (e.g. relevant and past misconduct) and attacks directed at motives (e.g. conflict of interest) would both reduce trust by challenging integrity and benevolence. Attacks directed at competence (e.g. our sloppy and education conditions) may reduce trust by challenging ability. One of our failed predictions was that an ad hominem attack (in the form of an accusation of misconduct) coupled with an explicit attack on the research itself would be more influential than an attack on the research alone. Using the Mayer et al. model, it may be that either of these two types of attacks may substantially reduce trust. If trust has already been reduced by one attack, a second attack may have no effect, simply because there is little trust left to erode. Another of our failed predictions was that ad hominem attacks (e.g. conflict of interest, past misconduct) would not lead to as much attitude change as attacks on the empirical foundation of the science claim (e.g. relevant misconduct or empirical). From the standpoint of the Mayer et al. model, it may be that the conflict of interest and past/relevant misconduct conditions would all reduce trust by challenging integrity and benevolence. For this reason, it would be expected that all of these attacks (i.e. conflict of interest, past misconduct, relevant misconduct) would impact participants similarly. Lastly, according to the Mayer et al. model, our education and sloppy conditions differ from the misconduct and conflict of interest conditions in that the former are related to the ability aspect of trust, while the latter are related to the benevolence and integrity aspects of trust. Because of this, the fact that the education and sloppy attacks were less effective than the conflict of interest, relevant and past misconduct attacks may indicate that attacks on ability are less effective than attacks on benevolence and integrity.

The current studies contribute to the literature in that they provide a relative measure of the impact of several types of ad hominem attacks, an empirical attack, and a combined attack on science claims. The results of the current studies confirm the findings of a number of studies, but are inconsistent with other studies. The finding that ad hominem attacks can successfully impact attitude about science claims is consistent with a number of studies that found a relationship between source credibility and attitude or trust in science claims. For instance, Lemanski and Villegas [55] used an experimental paradigm to find that a decrease in source credibility leads to a decrease in attitude towards claims found in pharmaceutical advertisements. Additionally, Yi, Yoon, Davis and Lee [59] found evidence that source expertise has a positive effect on perceived information quality. To the degree that misconduct and conflicts of interest are related to trust, the main findings of the current studies are consistent with findings related to the importance of trustworthiness in the risk analysis and public understanding of science literatures [13,15,24–27].

The current finding that knowledge of conflicts of interest has an impact on perceived source credibility and attitude in regards to science claims is also consistent with earlier research. Two studies [34,63] revealed that Australians place less faith in the claims of scientists funded by a private company in comparison to a scientist funded by a public university. Similarly, health advertisements attributed to unbiased sources were considered to be more credible [64], and British citizens are less likely to believe the research findings produced by scientists employed in the private sector [35]. Additionally, U.S. citizens are more confident in the safety of a new drug when the research on drug safety was funded by a university, rather than by a privately owned pharmaceutical company [54].

In the current studies, we found that the ad hominem attack directed at motives (conflict of interest) but not the ad hominem attacks directed at competence (sloppy and education) had an impact on attitude difference scores. While this finding is consistent with the Mayer et al. [9] framework of trust (in which ability, benevolence and integrity are distinct aspects of trust), the finding stands in contrast to Wolters, Steel, Lach, & Kloepfer [65], who found the quality of methodology employed by the researcher was much more important than source of funding in establishing researcher credibility. The inconsistency between the two studies may be explained by the different subjects (non-scientists vs. scientists) or by the different dependent measures employed (attitude difference scores vs. rating of importance).

The present research can be criticized because we used an attitude dependent measure, and attitude measures are not direct measures of choice behavior. However, Kahneman and colleagues [66] developed an argument that patterns of results found in tasks employing the dependent measures of choice and contingent valuation (CVM) ought to be found in tasks using attitude as a dependent measure. They claimed that choice is a special case of comparative valuation and argued that attitude and CVM responses are highly correlated and that the same things that affect dollar valuations (frames, anchors, etc.) tend to also affect attitudes as well (see also [67–69]). Additionally, Barnes and colleagues [61] demonstrated that the attitude difference dependent measure that we employed in the present study predicted choice outcomes of participants who were given the option to pick one prescription drug or another.

We concluded that ad hominem attacks mentioning misconduct and conflicts of interest have the same negative impact on attitudes of science claims as direct attacks on the empirical basis of those science claims. One could argue that we did not demonstrate that our participants were able to understand the methodological criticisms that were used as stimuli in the empirical condition (e.g. improper dependent measure, improper sample, etc.). Therefore, the true impact of challenging a source’s credibility may be far less than the impact of providing *understandable* criticisms of the research methodology. However, Klaczynski and colleagues [70–72] have shown that adolescents are able to detect methodological flaws such as the presence of a confounding variable, the use of an inappropriate dependent measure, the use of an inappropriate sample, and the use of an inappropriately small sample size. In the studies conducted by Klaczynski and colleagues, participants were provided with descriptions of studies and asked to report any flaws that they had noticed. In contrast to the Klaczynski paradigm, the methodological flaws in the current study were explicitly described as flaws. It therefore seems likely that most of our participants should have been able to understand the methodological criticisms employed as part of our stimuli.

No scientist is likely to debate the claim that flawed experimental design or deliberate misconduct should be expected to erode trust in research findings. However, some researchers addressing academic [73,74] and general [75] audiences have argued that conflicts of interest should be unrelated to the confidence placed in research findings. While the present research does not address the issue of what *ought* to influence scientists or the general public, we can report on what *does* influence non-scientists. The results of the current study indicate that laypersons significantly reduce their confidence in a claim due to knowledge of a conflict of interest. This has practical implications, as 91% of anti-vaccine websites explicitly claim that the bio-medical field is rife with conflicts of interests [76] and this communication tactic may play a part in the success of the anti-vaccine movement.

## Supporting information

S1 Table Initial claims for Experiments 1 & 2. (PDF)

S2 Table Summary of sequence of items within each questionnaire. (PDF)

S1 File List of science claims and attacks. (PDF)

S2 File Sample questionnaire. (PDF)

S3 File Experiment 1 data. (CSV)

S4 File Experiment 2 data. (CSV)
