# Supplementary material for: The effect of ad hominem attacks on the evaluation of claims promoted by scientists
Source: PLoS One. 2018 Jan 30;13(1):e0192025. doi: 10.1371/journal.pone.0192025 (PMC5790247; doi:10.1371/journal.pone.0192025)
Supplement: S1 Table — (PDF) [file pone.0192025.s001.pdf]

S1 Table. Initial claims for Experiments 1 & 2.

Claim #	Science Claim
**1**	According to Dr. Smith, a climate and energy researcher, nuclear power is just as inexpensive as power from coal and nuclear power has zero CO2 emissions.
**2**	According to Dr. Gumbilo and her group of ecologists at the University of Ohio, plane travel causes more global warming than car travel.
**3**	For the last couple of years, whale populations have been in sharp decline. According to marine biologist Dr. Rose, one reason why whale populations have been decreasing at such high rates is because the ultra high intensity sonar recently installed on US submarines is blasting the ear drums of whales so that they cannot hear. If whales can't find each other by sound, then they cannot mate with each other.
**4**	Dr. Doyle from the Children's Hospital of Pittsburgh claims that the chances of a child being diagnosed with Prudar-Wein syndrome decreases by over 20% if their diet includes niacin enriched baby food.
**5**	Dr. Zuirette of Brown University claims that the plastic used in fast food cups contains a chemical called oxalicide which has been recently linked with laryngeal cancer.
**6**	According to Dr. Johnson from the American Dermatological Association, Banana Boat brand tanning lotion blocks more UVA and UVB rays than the lotions made by their competitors.
**7**	According to Dr. Martinez at the University of Oklahoma, dibutylphthalate, a chemical used in Gold Bond foot powder, decreases the risk of some kinds of cancer.
**8**	Dr. Jensen, a botanist at the University of California, claims that the chemicals used in the Roundup brand herbicide are completely harmless to children.
**9**	Dr. Gray from New England Medical Center recently disclosed research findings that indicate that the consumption of redfish leads to an increased risk of lymphoblastic leukemia.
**10**	According to Dr. Cho's research, small amounts of the plastic in the plastic bottles used by some baby food companies leaches into the baby food. She claims that this plastic can act a carcinogen and that it is dangerous to children.
**11**	According to Dr. Hargrave of Haskins Engineering Lab, cars powered by hybrid engines will always use less fossil fuel than those powered by fuel cells.
**12**	Dr. Anderson of Timkin Labs has shown that steel brake pads doped with small amounts of nickel wear out faster than pads that are not doped with nickel.
