# Supplementary material for: The effect of ad hominem attacks on the evaluation of claims promoted by scientists
Source: PLoS One. 2018 Jan 30;13(1):e0192025. doi: 10.1371/journal.pone.0192025 (PMC5790247; doi:10.1371/journal.pone.0192025)
Supplement: S2 Table — (PDF) [file pone.0192025.s002.pdf]

S2 Table. Summary of sequence of items within each questionnaire.*

Questionnaire #1

Item#	Item type	Attack (if present)
1	Claim11	
2	Distractor1	
3	Distractor2	
4	Distractor3	
5	Claim5	
6	Claim6	Education
7	Claim8	Conflict of interest
8	Distractor4	
9	Distractor5	
10	Distractor6	
11	Claim3	
12	Distractor7	
13	Claim10	Sloppy
14	Distractor8	
15	Claim9	
16	Distractor9	
17	Distractor10	
18	Claim12	Empirical
19	Claim4	Past misconduct
20	Distracor11	
21	Claim7	
22	Distractor12	
23	Claim1	
24	Claim2	Relevant misconduct

*Each questionnaire contained 24 items. Twelve items were distractors, 12 were critical items. Critical items could be science claims in isolation or science claims followed by one of the six types of attack (e.g. empirical, relevant misconduct, etc.).

Questionnaire #2

Item#	Item type	Attack (if present)
1	Claim11	Empirical
2	Distractor1	
3	Distractor2	
4	Distractor3	
5	Claim5	
6	Claim6	Relevant misconduct
7	Claim8	
8	Distractor4	
9	Distractor5	
10	Distractor6	
11	Claim3	Past misconduct
12	Distractor7	
13	Claim10	
14	Distractor8	
15	Claim9	Education
16	Distractor9	
17	Distractor10	
18	Claim12	
19	Claim4	Conflict of interest
20	Distracor11	
21	Claim7	
22	Distractor12	
23	Claim1	Sloppy
24	Claim2	

Questionnaire #3

Item#	Item type	Attack (if present)
1	Claim11	
2	Distractor1	
3	Distractor2	
4	Distractor3	
5	Claim5	
6	Claim6	Conflict of interest
7	Claim8	Education
8	Distractor4	
9	Distractor5	
10	Distractor6	
11	Claim3	
12	Distractor7	
13	Claim10	Relevant misconduct
14	Distractor8	
15	Claim9	
16	Distractor9	
17	Distractor10	
18	Claim12	Past misconduct
19	Claim4	Empirical
20	Distracor11	
21	Claim7	
22	Distractor12	
23	Claim1	
24	Claim2	Sloppy

Questionnaire #4

Item#	Item type	Attack (if present)
1	Claim11	Relevant misconduct
2	Distractor1	
3	Distractor2	
4	Distractor3	
5	Claim5	Empirical
6	Claim6	
7	Claim8	
8	Distractor4	
9	Distractor5	
10	Distractor6	
11	Claim3	Sloppy
12	Distractor7	
13	Claim10	
14	Distractor8	
15	Claim9	Education
16	Distractor9	
17	Distractor10	
18	Claim12	
19	Claim4	
20	Distracor11	
21	Claim7	Conflict of interest
22	Distractor12	
23	Claim1	Past misconduct
24	Claim2	

Questionnaire #5

Item#	Item type	Attack (if present)
1	Claim11	
2	Distractor1	
3	Distractor2	
4	Distractor3	
5	Claim5	
6	Claim6	Sloppy
7	Claim8	Relevant misconduct
8	Distractor4	
9	Distractor5	
10	Distractor6	
11	Claim3	
12	Distractor7	
13	Claim10	Empirical
14	Distractor8	
15	Claim9	
16	Distractor9	
17	Distractor10	
18	Claim12	Education
19	Claim4	Conflict of interest
20	Distracor11	
21	Claim7	
22	Distractor12	
23	Claim1	
24	Claim2	Past misconduct

Questionnaire #6

Item#	Item type	Attack (if present)
1	Claim11	Empirical
2	Distractor1	
3	Distractor2	
4	Distractor3	
5	Claim5	Conflict of interest
6	Claim6	
7	Claim8	
8	Distractor4	
9	Distractor5	
10	Distractor6	
11	Claim3	Past misconduct
12	Distractor7	
13	Claim10	
14	Distractor8	
15	Claim9	Relevant misconduct
16	Distractor9	
17	Distractor10	
18	Claim12	
19	Claim4	
20	Distracor11	
21	Claim7	Sloppy
22	Distractor12	
23	Claim1	Education
24	Claim2	

Questionnaire #7

Item#	Item type	Attack (if present)
1	Claim11	
2	Distractor1	
3	Distractor2	
4	Distractor3	
5	Claim5	
6	Claim6	Relevant misconduct
7	Claim8	Sloppy
8	Distractor4	
9	Distractor5	
10	Distractor6	
11	Claim3	
12	Distractor7	
13	Claim10	Past misconduct
14	Distractor8	
15	Claim9	
16	Distractor9	
17	Distractor10	
18	Claim12	Conflict of interest
19	Claim4	Education
20	Distracor11	
21	Claim7	
22	Distractor12	
23	Claim1	
24	Claim2	Empirical

Questionnaire #8

Item#	Item type	Attack (if present)
1	Claim11	Past misconduct
2	Distractor1	
3	Distractor2	
4	Distractor3	
5	Claim5	Education
6	Claim6	
7	Claim8	
8	Distractor4	
9	Distractor5	
10	Distractor6	
11	Claim3	Empirical
12	Distractor7	
13	Claim10	
14	Distractor8	
15	Claim9	Sloppy
16	Distractor9	
17	Distractor10	
18	Claim12	
19	Claim4	
20	Distracor11	
21	Claim7	Relevant misconduct
22	Distractor12	
23	Claim1	Conflict of interest
24	Claim2	
