# Supplementary material for: The effect of ad hominem attacks on the evaluation of claims promoted by scientists
Source: PLoS One. 2018 Jan 30;13(1):e0192025. doi: 10.1371/journal.pone.0192025 (PMC5790247; doi:10.1371/journal.pone.0192025)
Supplement: S1 File — (PDF) [file pone.0192025.s003.pdf]

S1 File. List of science claims and attacks.

This document lists each of the 12 critical science claims along with the 6 attacks (empirical, conflict of interest, etc.) for each of those claims.

Science Claim 1

According to Dr. Smith, a climate and energy researcher, nuclear power is just as inexpensive as power from coal and nuclear power has zero CO_2 emissions.

1 Empirical

Dr. Smith's research related to nuclear power plants and CO_2 emissions only looks at direct CO_2 emissions. He fails to take into account substantial indirect CO_2 emissions associated with nuclear power plants.

1 Relevant Misconduct

Recently a team of investigators from the National Science Foundation's ethics committee found that Dr. Smith fabricated some of the data in his published research on nuclear power.

1 Past Misconduct

Recently a team of investigators from the National Science Foundation's ethics committee found that Dr. Smith fabricated some of the data in one of his earlier papers.

1 Conflict of Interest

Dr. Smith has been a paid consultant for a large nuclear power corporation for over 15 years.

1 Education

Dr. Smith received his advanced degree from a university with a reputation for having very low standards.

1 Sloppy

Many of the researchers in Dr. Smith's field feel that he is a sloppy researcher.

Science Claim 2
According to Dr. Gumbilo and her group of ecologists at the University of Ohio, plane travel causes more global warming than car travel.

2 Empirical
The research that was conducted by Dr. Gumbilo and her team of ecologists is flawed. She calculated the rate of auto emissions on January 10th and then multiplied that times 365 to get the amount of auto emissions per year. People drive less in the winter, so her figures probably underestimate the amount of auto emissions per year in the U.S.

2 Relevant Misconduct
Recently a team of investigators from the National Science Foundation's ethics committee found that Dr. Gumbilo fabricated some of the data in her published research on global warming.

2 Past Misconduct
Recently a team of investigators from the National Science Foundation's ethics committee found that Dr. Gumbilo fabricated some of the data in one of her earlier papers.

2 Conflict of Interest
Dr. Gumbilo and her group of ecologists have been paid consultants for a major automaker for the past 11 years.

2 Education
Dr. Gumbilo received her advanced degree from a university with a reputation for having very low standards.

2 Sloppy
Many of the researchers in Dr. Gumbilo's field feel that she is a sloppy researcher.

Science Claim 3

For the last couple of years, whale populations have been in sharp decline. According to marine biologist Dr. Rose, one reason why whale populations have been decreasing at such high rates is because the ultra high intensity sonar recently installed on US submarines is blasting the ear drums of whales so that they cannot hear. If whales can't find each other by sound, then they cannot mate with each other.

3 Empirical

Dr. Rose's research on the impact of high intensity sonar was done within 10 miles of the coast. Many whales spend time far from US coastal waters and they may not be affected by submarine sonar.

3 Relevant misconduct

Recently a team of investigators from the National Science Foundation's ethics committee found that Dr. Rose fabricated some of the data in his published research the effect of sonar on whales.

3 Past misconduct

Recently a team of investigators from the National Science Foundation's ethics committee found that Dr. Rose fabricated some of the data in one of his earlier papers.

3 Conflict of Interest

Dr. Rose is a paid staff member for PETA (People for the Ethical Treatment of Animals).

3 Education

Dr. Rose received his advanced degree from a university with a reputation for having very low standards.

3 Sloppy

Many of the researchers in Dr. Rose's field feel that he is a sloppy researcher.

Science Claim 4
Dr. Doyle from the Children's Hospital of Pittsburgh claims that the chances of a child being diagnosed with Prudar-Wein syndrome decreases by over 20% if their diet includes niacin enriched baby food.

4 Empirical
Dr. Doyle's research on the effect of niacin on Prudar-Wein syndrome only included children ages 28 to 34 months of age. However, Prudar-Wein syndrome is normally diagnosed by 18 months of age.

4 Relevant misconduct
Recently a team of investigators from the National Science Foundation's ethics committee found that Dr. Doyle fabricated some of the data in her published research on Prudar-Wein syndrome.

4 Past misconduct
Recently a team of investigators from the National Science Foundation's ethics committee found that Dr. Doyle fabricated some of the data in one of her earlier papers.

4 Conflict of Interest
Dr. Doyle is an employee of the only baby food company that adds niacin to its baby food.

4 Education
Dr. Doyle received her advanced degree from a university with a reputation for having very low standards.

4 Sloppy
Many of the researchers in Dr. Doyle's field feel that she is a sloppy researcher.

Science Claim 5
Dr. Zuirette of Brown University claims that the plastic used in fast food cups contains a chemical called oxalicide which has been recently linked with laryngeal cancer.

5 Empirical
The research that was conducted by Dr. Zuirette involved a chemical test that is known to produce many false positives. In other words, the test will sometimes indicate that a chemical is present, when it is not.

5 Relevant misconduct
Recently a team of investigators from the National Science Foundation's ethics committee found that Dr. Zuirette fabricated some of the data in his published research on oxalicide.

5 Past misconduct
Recently a team of investigators from the National Science Foundation's ethics committee found that Dr. Zuirette fabricated some of the data in one of his earlier papers.

5 Conflict of Interest
Dr. Zuirette has been paid for the last 6 years by Stanton Plastics Co. which makes cups that do not contain oxalicide.

5 Education
Dr. Zuirette received his advanced degree from a university with a reputation for having very low standards.

5 Sloppy
Many of the researchers in Dr. Zuirette's field feel that he is a sloppy researcher.

Science Claim 6
According to Dr. Johnson from the American Dermatological Association, Banana Boat brand tanning lotion blocks more UVA and UVB rays than the lotions made by their competitors.

6 Empirical
When Dr. Johnson tested the tanning lotion from Banana Boat, she used a sample that had not reached its expiration date. However, when testing tanning lotion made by other companies, she used samples that were past their expiration dates.

6 Relevant misconduct
Recently a team of investigators from the National Science Foundation's ethics committee found that Dr. Johnson fabricated some of the data in her published research on Banana Boat tanning lotion.

6 Past misconduct
Recently a team of investigators from the National Science Foundation's ethics committee found that Dr. Johnson fabricated some of the data in one of her earlier papers.

6 Conflict of Interest
Each year for the last 15 years Banana Boat Inc. has provided Dr. Johnson all expenses paid vacations to Hawaii.

6 Education
Dr. Johnson received her advanced degree from a university with a reputation for having very low standards.

6 Sloppy
Many of the researchers in Dr. Johnson's field feel that she is a sloppy researcher.

Science Claim 7
According to Dr. Martinez at the University of Oklahoma, dibutylphthalate, a chemical used in Gold Bond foot powder, decreases the risk of some kinds of cancer.

7 Empirical
Dr. Martinez's research on Gold Bond foot powder failed to employ a control group with which to compare the cancer rates of his foot powder using group.

7 Relevant misconduct
Recently a team of investigators from the National Science Foundation's ethics committee found that Dr. Martinez fabricated some of the data in his published research on Gold Bond foot powder.

7 Past misconduct
Recently a team of investigators from the National Science Foundation's ethics committee found that Dr. Martinez fabricated some of the data in one of his earlier papers.

7 Conflict of Interest
Dr. Martinez has been a paid consultant for Chattam, Inc. (the company that makes Gold Bond foot powder) for over 8 years.

7 Education
Dr. Martinez received his advanced degree from a university with a reputation for having very low standards.

7 Sloppy
Many of the researchers in Dr. Martinez's field feel that he is a sloppy researcher.

Science Claim 8
Dr. Jensen, a botanist at the University of California, claims that the chemicals used in the Roundup brand herbicide are completely harmless to children.
8 Empirical
Dr. Jensen only tested children exposed to mild doses of Roundup. Many consumers use larger quantities of the product, therefore her tests may not reflect actual exposure to the chemical.
8 Relevant misconduct
Recently a team of investigators from the National Science Foundation's ethics committee found that Dr. Jensen fabricated some of the data in her published research on Roundup.
8 Past misconduct
Recently a team of investigators from the National Science Foundation's ethics committee found that Dr. Jensen fabricated some of the data in one of her earlier papers.
8 Conflict of Interest
Dr. Jensen is head of the research and development branch of the company that manufactures Roundup herbicide.
8 Education
Dr. Jensen received her advanced degree from a university with a reputation for having very low standards.
8 Sloppy
Many of the researchers in Dr. Jensen's field feel that she is a sloppy researcher.

Science Claim 9

Dr. Gray from New England Medical Center recently disclosed research findings that indicate that the consumption of redfish leads to an increased risk of lymphoblastic leukemia.

9 Empirical

The sample that Dr. Gray used was small (less than 60 subjects). A much larger group of people should be studied before any conclusions can be made about the effect of eating redfish.

9 Relevant misconduct

Recently a team of investigators from the National Science Foundation's ethics committee found that Dr. Gray fabricated some of the data in his published research on redfish.

9 Past misconduct

Recently a team of investigators from the National Science Foundation's ethics committee found that Dr. Gray fabricated some of the data in one of his earlier papers.

9 Conflict of Interest

Dr. Gray's research was paid for by the sometimes extremist Marine Life Protection Organization.

9 Education

Dr. Gray received his advanced degree from a university with a reputation for having very low standards.

9 Sloppy

Many of the researchers in Dr. Gray's field feel that he is a sloppy researcher.

Science Claim 10
According to Dr. Cho's research, small amounts of the plastic in the plastic bottles used by some baby food companies leaches into the baby food. She claims that this plastic can act a carcinogen and that it is dangerous to children.

10 Empirical
Dr. Cho heated the baby bottles to 120 deg Fahrenheit and *then* tested the food to see if the plastic had leached into the food. While it is true that the label on the bottles reads "store between 50 and 120 degrees Fahrenheit", it is unlikely that most people will actually store the bottles someplace that is that hot.

10 Relevant misconduct
Recently a team of investigators from the National Science Foundation's ethics committee found that Dr. Cho fabricated some of the data in her published research on plastic bottles.

10 Past misconduct
Recently a team of investigators from the National Science Foundation's ethics committee found that Dr. Cho fabricated some of the data in one of her earlier papers.

10 Conflict of Interest
Dr. Cho's brother works at a company that makes glass containers. If the baby food companies that used plastic bottles switched to glass, Dr. Cho's brother would get more business and make more money.

10 Education
Dr. Cho received her advanced degree from a university with a reputation for having very low standards.

10 Sloppy
Many of the researchers in Dr. Cho's field feel that she is a sloppy researcher.

Science Claim 11
According to Dr. Hargrave of Haskins Engineering Lab, cars powered by hybrid engines will always use less fossil fuel than those powered by fuel cells.
11 Empirical
When Dr. Hargrave compared the amount of fossil fuel used by fuel cell cars compared to hybrid cars, he compared the fuel cell car with the worst mileage to the hybrid car with the best mileage. This would make hybrid engines look better than they should.
11 Relevant misconduct
Recently a team of investigators from the National Science Foundation’s ethics committee found that Dr. Hargrave fabricated some of the data in his published research on hybrid engines.
11 Past misconduct
Recently a team of investigators from the National Science Foundation’s ethics committee found that Dr. Hargrave fabricated some of the data in one of his earlier papers.
11 Conflict of Interest
Dr. Hargrave is employed as a consultant by Shiba, the company that manufactures hybrid engines for vehicles like the Toyota Prius.
11 Education
Dr. Hargrave received his advanced degree from a university with a reputation for having very low standards.
11 Sloppy
Many of the researchers in Dr. Hargrave’s field feel that he is a sloppy researcher.

Science Claim 12
Dr. Anderson of Timkin Labs has shown that steel brake pads doped with small amounts of nickel wear out faster than pads that are not doped with nickel.
12 Empirical
While it is true that, in theory, doping steel with nickel will make it wear out faster, it is also true that adding nickel prevents the brakes from rusting. Dr. Anderson failed to take the issue of rust into account.
12 Relevant misconduct
Recently a team of investigators from the National Science Foundation's ethics committee found that Dr. Anderson fabricated some of the data in published her research on brakes.
12 Past misconduct
Recently a team of investigators from the National Science Foundation's ethics committee found that Dr. Anderson fabricated some of the data in one of her earlier papers.
12 Conflict of Interest
Dr. Anderson's family owns the only auto parts manufacturing company that still manufactures brake pads made with steel that does not include any nickel.
12 Education
Dr. Anderson received her advanced degree from a university with a reputation for having very low standards.
12 Sloppy
Many of the researchers in Dr. Anderson's field feel that she is a sloppy researcher.
