# Supplementary material for: The effect of ad hominem attacks on the evaluation of claims promoted by scientists
Source: PLoS One. 2018 Jan 30;13(1):e0192025. doi: 10.1371/journal.pone.0192025 (PMC5790247; doi:10.1371/journal.pone.0192025)
Supplement: S2 File — (PDF) [file pone.0192025.s004.pdf]

S2 File. Sample questionnaire.

This document contains questionnaire #1 (there are one eight questionnaire variants) used in Experiments 1 and 2. The questionnaire starts on the next page.

Items in the questionnaire that are in grey font are distractor items. Items that are in black font are the 12 critical claims.

The demographic items for Experiment 1 are located on page 11 of this document.

The demographic items for Experiment 2 are located on pages 12 and 13 of this document.

Instructions

For each issue, please *carefully* read all the material presented to you. The paragraph on the left will promote a particular hypothesis. The paragraph on the right (if present) will present additionally information. After you have read the material for a particular issue, indicate your feelings about the way you feel about the hypothesis promoted in the paragraph on the ***left.***

Example issue: the cause of the common cold

Claim A

According to one source…
The common cold is caused by a virus.

According to another source…
The common cold is caused by going outside in the winter with wet hair.

Favor Claim A 1 2 3 4 5 6 Oppose Claim A

In the example above you would circle a
1 if you strongly favored the virus hypothesis
2 if you moderately favored the virus hypothesis
3 if you somewhat favored the virus hypothesis
4 if you somewhat opposed the virus hypothesis
5 if you moderately opposed the virus hypothesis
6 if you strongly opposed the virus hypothesis

In the example above, the “2” is circled indicating that the person moderately favored the virus hypothesis.

You should not skip any issues. Please circle a number in the scale below *each* issue.

Section 1

Claim #1
According to one source…
According to Dr. Hargrave of Haskins Engineering Lab, cars powered by hybrid engines will always use less fossil fuel than those powered by fuel cells.

Favor Claim #1 **1** **2** **3** **4** **5** **6** **Oppose Claim #1**

Claim #2
According to one source…
Clusters of prions in the brain cause Lou Gehrig's disease.

Favor Claim #2 **1** **2** **3** **4** **5** **6** **Oppose Claim #2**

Claim #3
According to one source…
Direct electrical stimulation of the splanchnic nerve is effective at treating cases of severe depression.

According to another source…
Over the last decade, a series of scientific articles have been published on the effects of electrical stimulation of the splanchnic nerve and 9% of those papers concluded that direct electrical stimulation of the splanchnic nerve does effectively treat cases of severe depression.

Favor Claim #3 **1** **2** **3** **4** **5** **6** **Oppose Claim #3**

Claim #4
According to one source…
Magnesium diboride becomes a superconductor at -126 degrees Celsius.

Favor Claim #4 1 2 3 4 5 6 Oppose Claim #4

Claim #5
According to one source…
Dr. Zuirette of Brown University claims that the plastic used in fast food cups contains a chemical called oxalicide which has been recently linked with laryngeal cancer.

.

Favor Claim #5 1 2 3 4 5 6 Oppose Claim #5

Claim #6
According to one source…
According to Dr. Johnson from the American Dermatological Association, Banana Boat brand tanning lotion blocks more UVA and UVB rays than the lotions made by their competitors.

According to another source…
Dr. Johnson received her advanced degree from a university with a reputation for having very low standards.

Favor Claim #6 1 2 3 4 5 6 Oppose Claim #6

Claim #7

According to one source…
Dr. Jensen, a botanist at the University of California, claims that the chemicals used in the Roundup brand herbicide are completely harmless to children.

According to another source…
Dr. Jensen is head of the research and development branch of the company that manufactures Roundup herbicide.

Favor Claim #7 1 2 3 4 5 6 Oppose Claim #7

Claim #8

According to one source…
The decline of the population of fresh water perch in Lake Erie is due to competition from snipe eels, a non-native species that was introduced to the Lake in the 1990s.

According to another source…
Several freshwater marine biologists have publicly weighed in on the fresh water perch issue and 77% of them claim that snipe eels are the cause for the decline of the perch population.

Favor Claim #8 1 2 3 4 5 6 Oppose Claim #8

Claim #9

According to one source…
A new drug, Amgonalen, effectively treats schizoid personality disorder.

According to another source…
Researchers in both Europe and the U.S. have been investigating Amgonalen and 84% of those researchers have found support for the claim that Amgonalen effectively treats schizoid personality disorder.

Favor Claim #9 1 2 3 4 5 6 Oppose Claim #9

Claim #10
According to one source…
A team of African biologists found that creating a series of medium sized animal preserves throughout a rainforest drastically decreases the poaching of apes and monkeys by hunters.

Favor Claim #10 1 2 3 4 5 6 Oppose Claim #10

Claim #11
According to one source…
For the last couple of years, whale populations have been in sharp decline. According to marine biologist Dr. Rose, one reason why whale populations have been decreasing at such high rates is because the ultra high intensity sonar recently installed on US submarines is blasting the ear drums of whales so that they cannot hear. If whales can't find each other by sound, then they cannot mate with each other.

Favor Claim #11 1 2 3 4 5 6 Oppose Claim #11

Claim #12
According to one source…
According to a research team at U.C.L.A., infants that consumed formula that was supplemented with lutein had higher I.Q. scores at age 10 compared to infants that consumed regular formula. Therefore, the nutritional supplement lutein, when given to infants enhances brain development.

Favor Claim #12 1 2 3 4 5 6 Oppose Claim #12

Claim #13

According to one source…
According to Dr. Cho's research, small amounts of the plastic in the plastic bottles used by some baby food companies leaches into the baby food. She claims that this plastic can act a carcinogen and that it is dangerous to children.

According to another source…
Many of the researchers in Dr. Cho's field feel that she is a sloppy researcher.

Favor Claim #13 1 2 3 4 5 6 Oppose Claim #13

Claim #14

According to one source…
According to two prominent psychiatrists, a new drug, Prevadarin, effectively treats obsessive compulsive disorder.

According to another source…
Researchers at a number of universities tested Prevadarin for its effect on obsessive compulsive disorder and 17% of those studies showed that Prevadarin decreased the symptoms of the disorder.

Favor Claim #14 1 2 3 4 5 6 Oppose Claim #14

Claim #15

According to one source…
Dr. Gray from New England Medical Center recently disclosed research findings that indicate that the consumption of redfish leads to an increased risk of lymphoblastic leukemia.

Favor Claim #15 1 2 3 4 5 6 Oppose Claim #15

Claim #16

According to one source…
A team of researchers at City College of New York concluded from the rock samples they analyzed that 34,000 years ago Manhattan was not an island but was part of the mainland.

According to another source…
A number of geology papers on the geological history of Manhattan have been published and 69% of them agree with the researchers at City College of New York that 34,000 years ago Manhattan was not an island but was part of the mainland.

Favor Claim #16 1 2 3 4 5 6 Oppose Claim #16

Claim #17

According to one source…
A group of biologists at Washington State University recently compared the DNA of jaguars with that of other great cats. They found the highest degree of similarity between jaguars and lynxes and concluded that the lynx is the closest relative of the jaguar.

Favor Claim #17 1 2 3 4 5 6 Oppose Claim #17

Claim #18

According to one source…
Dr. Anderson of Timkin Labs has shown that steel brake pads doped with small amounts of nickel wear out faster than pads that are not doped with nickel.

According to another source…
While it is true that, in theory, doping steel with nickel will make it wear out faster, it is also true that adding nickel prevents the brakes from rusting. Dr. Anderson failed to take the issue of rust into account.

Favor Claim #18 1 2 3 4 5 6 Oppose Claim #18

Claim #19

According to one source…
Dr. Doyle from the Children's Hospital of Pittsburgh claims that the chances of a child being diagnosed with Prudar-Wein syndrome decrease by over 20% if their diet includes niacin enriched baby food.

According to another source…
Recently a team of investigators from the National Science Foundation's ethics committee found that Dr. Doyle fabricated some of the data in one of her earlier papers.

Favor Claim #19 1 2 3 4 5 6 Oppose Claim #19

Claim #20

According to one source…
A team of astronomers at the Chandra X-ray Observatory found that the sun emits 30 times more X-rays than most other stars of the same size and age.

According to another source…
Astronomers using X-ray telescopes have analyzed the X-ray output of the sun and compared it to similar stars and 24% of the studies confirmed that the sun has an X-ray output 30 times higher than similar stars.

Favor Claim #20 1 2 3 4 5 6 Oppose Claim #20

Claim #21

According to one source…
According to Dr. Martinez at the University of Oklahoma, dibutylphthalate, a chemical used in Gold Bond foot powder, decreases the risk of some kinds of cancer.

Favor Claim #21 1 2 3 4 5 6 Oppose Claim #21

Claim #22
According to one source…
Laparoscopic surgery results in high levels of post operative infection when the technique is used to remove a person's appendix.

Favor Claim #22 1 2 3 4 5 6 Oppose Claim #22

Claim #23
According to one source…
According to Dr. Smith, a climate and energy researcher, nuclear power is just as inexpensive as power from coal and nuclear power has zero CO_2 emissions.

Favor Claim #23 1 2 3 4 5 6 Oppose Claim #23

Claim #24
According to one source…
According to Dr. Gumbilo and her group of ecologists at the University of Ohio, plane travel causes more global warming than car travel.

According to another source…
Recently a team of investigators from the National Science Foundation's ethics committee found that Dr. Gumbilo fabricated some of the data in her published research on global warming.

Favor Claim #24 1 2 3 4 5 6 Oppose Claim #24

[Demographic items for Experiment 1]

Sex (circle one)
F M

Age

_____

Class status

a. High school senior
b. College freshman
b. College sophomore
c. College junior
d. College senior
e. Bachelor's degree (or more) completed

If applicable, what is the course title for which you are completing this survey?
(e.g. Introduction to Psychology, Methods & statistics, etc.)

____

University or college attended _________________

Questionnaire 1

[Demographic items for Experiment 2]

Age _____

Sex (circle one)
F M

Highest Level of Education Attained

- Some high school
- High school diploma or equivalent
- Some college
- Bachelor’s degree
- Some graduate school
- Master’s Degree
- Doctoral Degree

In which state do you live? _______

How knowledgeable are you in regards to science?

- Not very knowledgeable
- Somewhat knowledgeable
- Moderately knowledgeable
- Very knowledgeable

What is your total household income?

- Less than $10,000
- $10,000 - $19,999
- $20,000 - $29,999
- $30,000 - $39,999
- $40,000 - $49,999
- $50,000 - $59,999
- $60,000 - $69,999
- $70,000 - $79,999
- $80,000 - $89,999
- $90,000 - $99,999
- $100,000 or more

How would you describe yourself? (Please select the one option that best describes you)

- American Indian or Alaska Native
- Hawaiian or Other Pacific Islander
- Asian or Asian American
- Black or African American
- Hispanic or Latino
- Non-Hispanic White

Marital status

- Married
- Divorced
- Widowed
- Separated
- Never been married
- A member of an unmarried couple
